# Validity of Machine Learning in Detecting Complicated Appendicitis in a Resource-Limited Setting: Findings from Vietnam

**DOI:** 10.1155/2023/5013812

**Published:** 2023-04-14

**Authors:** Tuong-Anh Phan-Mai, Truc Thanh Thai, Thanh Quoc Mai, Kiet Anh Vu, Cong Chi Mai, Dung Anh Nguyen

**Affiliations:** ^1^General Surgery Department, Nhan dan Gia Dinh Hospital, 1 No Trang Long Street, Ward 7, Binh Thanh District, Ho Chi Minh City, Vietnam; ^2^Department of Medical Statistics and Informatics, University of Medicine and Pharmacy at Ho Chi Minh City, 217 Hong Bang Street, Ward 11, District 5, Ho Chi Minh City, Vietnam; ^3^Planning Department, Nhan dan Gia Dinh Hospital, 1 No Trang Long Street, Ward 7, Binh Thanh District, Ho Chi Minh City, Vietnam

## Abstract

**Background:**

Complicated appendicitis, a potentially life-threatening condition, is common. However, the diagnosis of this condition is mainly based on physician's experiences and advanced diagnostic equipment. This study built and validated machine learning models to facilitate the detection of complicated appendicitis.

**Methods:**

A retrospective cohort study was conducted based on medical charts of all patients undergoing a laparoscopic appendectomy at a city hospital during 2016-2020. The synthetic minority over-sampling technique (SMOTE) was used to adjust for the imbalance. Multiple classification approaches were used to train and validate models including support vector machine (SVM), decision tree (DT), K-nearest neighbor (KNN), logistic regression (LR), artificial neural network (ANN), and gradient boosting (GB).

**Results:**

Among 1,950 patients included in the data analysis, there were 483 patients identified as having complicated appendicitis (24.8%). Based on data without SMOTE adjustment for imbalance, the accuracy levels and AUCs were high in all models using different parameters, ranging from 0.687 to 0.815. After adjusting for imbalance data using SMOTE, AUC and accuracy levels in the models using imbalance adjusted data were higher. Of these, the GB had all AUC and accuracy values of approximately 0.8 or more in both adjusted and unadjusted data.

**Conclusions:**

Machine learning approaches including SVM, DT, logistic, KNN, ANN, and GB have a high level of validity in classifying patients with complicated appendicitis and patients without complicated appendicitis. Among these, GB had the highest level of validity and should be used or further validated. Our study indicates the beneficial potentials of machine learning techniques in a clinical setting in general and in the diagnosis of complicated appendicitis in particular.

## 1. Introduction

Appendicitis is one of the most common emergency gastroenteric diseases. Previous studies have reported that about 7%-10% of emergency cases had abdominal pain, almost all of whom had right lower abdominal quadrant pain and were subsequently diagnosed as appendicitis [1]. However, the prevalence of appendicitis ranges widely across countries, for example, 206 cases per 100,000 person-years in South Africa, 100 cases per 100,000 person-years in America, and 206 cases per 100,000 person-years in Korea [2]. Importantly, complicated appendicitis which is considered a life-threatening condition is also common. The prevalence of appendicitis with perforation ranges from 16% to 40% and is higher in people aged over 50 years (from 55% to 70%) [3]. Patients with appendicitis who had perforation or phlegmon have a significant higher mortality rate than those without perforation. Moreover, although appendectomy is still one of the most common treatments, many recent studies have provided evidence of positive outcomes of nonoperative treatment in some cases [4]. Therefore, early and timely diagnosis of complicated appendicitis plays a vital role in choosing the proper treatment and minimizing further serious complications.

To date, several scoring systems have been developed and validated to help screen and diagnose acute appendicitis and to predict complicated appendicitis such as the Alvarado score, ARIs, RIPASA, and APSI [5–7]. However, these tools require data which are not always available, especially in resource-limited settings. For example, to diagnose appendicitis using the APSI, a CT scan is required. Although CT scan has been considered a gold standard for diagnosis of appendicitis, this technique is not feasible in primary care settings in the absence of specialists and equipment while the risk of radiation exposure from CT scan is still controversial [7]. Other tools such as the Alvarado requires clinical symptoms which may be misdiagnosed or subjectively identified by physicians, leading to both false positive and false negative appendicitis. Therefore, the use of basic information such as blood test and ultrasound to early screen complicated appendicitis is beneficial. Fortunately, the presence of artificial intelligence and machine learning (ML) techniques can make this idea feasible and practical as reported in previous studies [8–11].

The advantages of machine learning techniques in the diagnosis of diseases have been well documented including its application in appendicitis. First, ML helps physicians objectively and correctly examine different types of inflammation of the appendix. This is because the clinical signs and symptoms are not always specific, and physicians have to combine many information such as health status, signs, and laboratory tests to support their diagnosis which depends significantly on their experiences. In this regard, once trained and tested, ML can make a diagnosis with a high level of reliability in a timely period. In addition, physicians and surgeons can take advantage of the results provided by ML to decide the most appropriate treatments for patients, which helps decrease the risk of adverse events of appendectomy, either laparoscopic appendectomy or open appendectomy. For acute appendicitis, many recent studies reported that antibiotic and nonoperative treatment result in similar treatment outcomes compared to appendectomy [12]. Finally, the application of ML in screening and diagnosis of complicated appendicitis helps medical systems and specialists avoid overload, particularly higher tier hospitals because a certain number of patients can be diagnosed and treated at primary care settings.

Therefore, the aim of this study was to examine the validity of ML in detecting complicated appendicitis at a tertiary hospital in Ho Chi Minh City, Vietnam. Findings from this study provide scientific evidence of whether or not ML can be used in other resource-limiting settings.

## 2. Materials and Methods

### 2.1. Study Design and Settings

A retrospective cohort study was conducted based on medical charts of all patients who had a laparoscopic appendectomy at the Department of Gastrointestinal Surgery in Gia Dinh People Hospital in Ho Chi Minh City, Vietnam, from 2016 to 2020. This hospital is a city hospital with 18 specialty departments. Each year, approximately 1000 patients have appendicitis and subsequently have laparoscopic appendectomy which is a standard of treatment at the hospital. During the study period, all medical records of these patients including all clinical and subclinical information as well as surgery reports were collected. This study was approved by the Ethical Committee at Gia Dinh People Hospital (approval number: 16/NDGĐ-HĐĐĐ).

### 2.2. Measurement

At the study hospital, patient's data are stored in electronic medical records. However, similar to other resource-limited hospitals in Vietnam, this electronic medical record system is not perfect. Although identification information of all patients is available in such a system, detail data are not always available. In our study, about 50% of patients' data were extracted from a hard copy of their medical records. Data were extracted from both electronic and hard copy medical records, including demographic characteristics (i.e., age and gender), blood tests, and ultrasound. Blood tests consisted of total white blood cell count (cells per cubic millimeter—cells/mm^3^), granulocyte count (cells/mm^3^), lymphocyte count (cells/mm^3^), and C-reactive protein test (mg/L). The diameter of the appendix, extraluminal free air, periappendiceal fluid, and abscess was recorded through ultrasound results. The ultrasound data were based on the conclusions noted in the medical records, not from the ultrasound images. The diagnosis of complicated appendicitis was confirmed based on inflammation of the appendix including perforation (appendiceal rupture), phlegmon, and generalized peritonitis (accounting for appendicitis). These conditions were identified based on standard surgical reports. The data structure is summarized in Table 1.

### 2.3. Data Analysis

Among 4,242 patients who underwent a laparoscopic appendectomy at the hospital during 2016–2020, there were missing data in either blood tests, ultrasound results, or surgical reports in 1217 patients and thus were excluded from the analysis. Data of 1,950 patients included in the analysis were randomly divided into two parts: 70% for training and 30% for testing. Ideally, the data used for both training and testing should have one control (i.e., no complicated appendicitis) per case (i.e., complicated appendicitis) which results in a prevalence of complicated appendicitis of 50%. In fact, the prevalence of complicated appendicitis was much lower, and thus, the imbalance of the real data might affect the validity of the models fitted. Therefore, the synthetic minority over-sampling technique (SMOTE) was used to adjust for the imbalance [13, 14]. In this study, both imbalanced and balanced data based on SMOTE were used to build and evaluate models. Because the features included were in different ranges, normalization was also applied using the standard formula: *x*_norm_ = (*x*–*x*_min_)/(*x*_max_–*x*_min_). Although principal component analysis is beneficial for the situations where there are high-dimensional data, we also used this approach to check the 12 features included. Multiple classification approaches were used to train models including support vector machine (SVM), decision tree (DT), K-nearest neighbor (KNN), logistic regression (LR), artificial neural network (ANN), and gradient boosting (GB). These approaches are commonly used in previous studies in disease classification [15]. The testing and evaluation of these models were based on the area under the curve (AUC) and the accuracy score. All data analyses were conducted using Python.

## 3. Results

**Figure 1:**
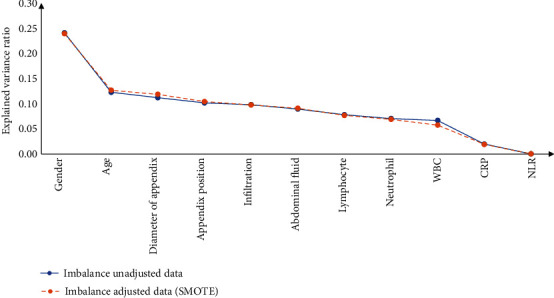
Principal component analysis of all features included.

Among 4,242 patients who underwent a laparoscopic appendectomy at the hospital during 2016–2020, there were missing data in either blood tests, ultrasound results, or surgical reports in 1217 patients and thus were excluded from the analysis. Among 1,950 patients included in data analysis, 45.0% (*n* = 678) were males and the mean age was 37.3 (SD = 15.9) years. Based on surgery reports, there were 483 patients identified as having complicated appendicitis (24.8%). There were significant differences between patients with and patients without complicated appendicitis in most characteristics measured including age, gender, neutrophil, C-reactive protein, diameter of appendix, and appendix position (Table 2).  Figure 1 presents results from the principal component analysis. Although a few features did not have high levels of explanation, these features have been proven to be important characteristics of acute appendicitis in clinical practice. Therefore, all these 12 features were kept in further analysis.

Table 3 presents results after building and evaluating different models including SVM, DT, logistic, KNN, ANN, and GB. Based on data without SMOTE adjustment for imbalance, the accuracy levels and AUCs were high in all models using different parameters, ranging from 0.687 to 0.815. After adjusting for imbalance data using SMOTE, AUC and accuracy levels in the model using imbalance adjusted data were higher (Table 3).

**Figure 2:**
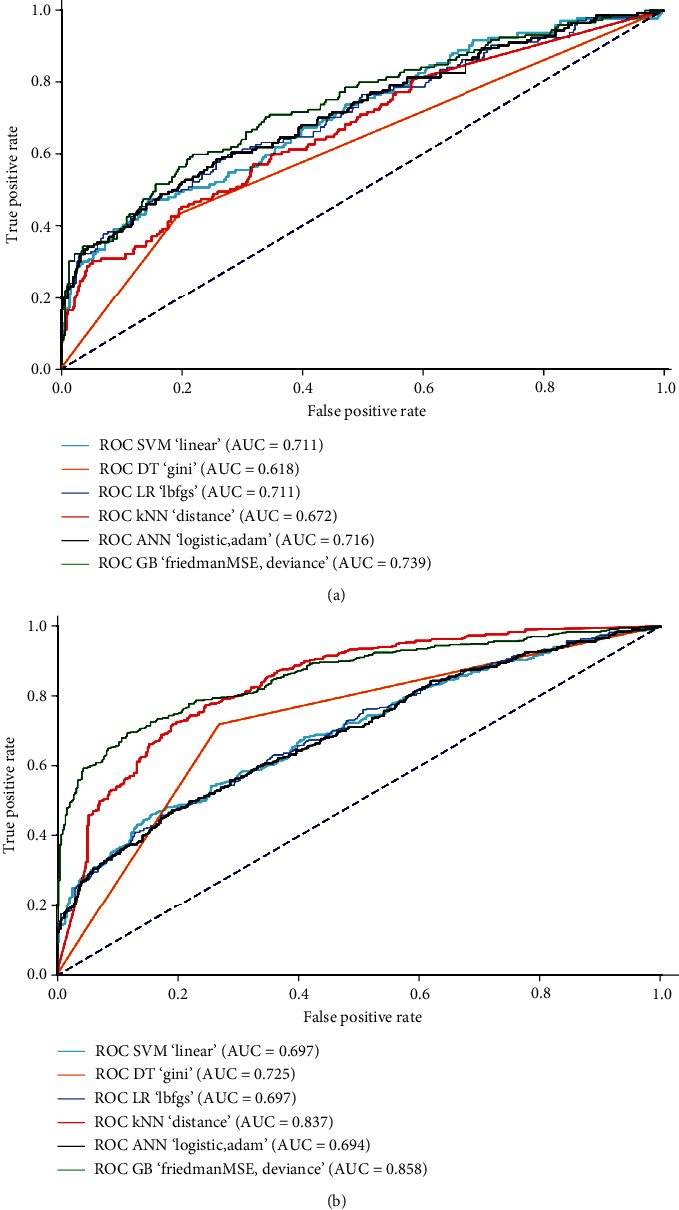
ROC curves of optimal models in classifying patients with and without complicated appendicitis. (a) Imbalance unadjusted data. (b) Imbalance adjusted data (SMOTE).

**Figure 3:**
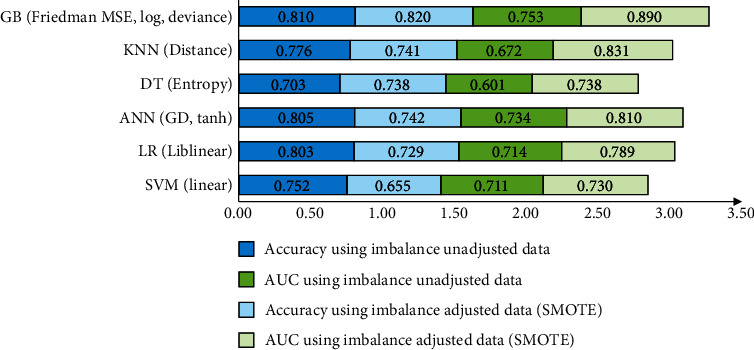
Optimal parameters for each model trained and evaluated for classification of patients with and without complicated appendicitis.

Based on the k-fold validation, optimal parameters were selected and the results are presented in Figures 2 and 3. All models with optimal parameters had good to excellent ability to classify patients with complicated appendicitis and patients without complicated appendicitis. Of these, the GB had all AUC and accuracy values of approximately 0.8 or more in both adjusted and unadjusted data.

## 4. Discussion

This study was among the very first in Vietnam to evaluate ML approaches in clinical settings and the first in the classification of complicated appendicitis. In a population with a relatively low prevalence of complicated appendicitis, the ML approaches including SVM, DT, logistic, KNN, ANN, and GB had good to excellent performance in classifying patients with complicated appendicitis and patients without complicated appendicitis.

The prevalence of patients with complicated appendicitis in this study was the same as that reported in previous studies where around 25% of the 300,000 cases of appendectomy each year had complicated appendicitis based on CT scan [16]. Other studies in Vietnam have reported the percentage of complicated appendicitis of 30%-40% [17, 18]. In particular, Van Tan illustrated that 31% of complicated appendicitis cases were determined during the surgery, while Quoc Anh et al. found a percentage of complicated appendicitis of 38% based on operative reports and pathology results. One possible explanation for our findings is the study population. For example, while patients in our study were those who underwent laparoscopic appendectomies, patients from other studies were those who underwent either laparoscopic or open appendectomies. Because open appendectomies are normally indicated for patients with severely complicated appendicitis, the prevalence of complicated appendicitis is expected to be higher. Moreover, although a CT scan can be considered the gold standard in the diagnosis of complicated appendicitis, the prevalence of complicated appendicitis found during operative or based on operative reports may be relatively different. In our study, this imbalance distribution of complicated appendicitis versus noncomplicated appendicitis was adjusted in further analysis.

The inclusion of features in ML approaches is important and contributes significantly to the overall performance of the models. In our study, features used in building machine learning models have been well documented in clinical literature and were confirmed from our data. Complicated appendicitis was found in old patients with high neutrophil and lymphocyte, neutrophil to lymphocyte ratio (NLR), CRP, diameter, and position of the appendix (from abdominal ultrasound). Particularly, a retrospective study with 498 patients who had appendectomy illustrated that CRP was great validity of detecting and distinguishing between complicated and uncomplicated appendicitis [19]. Moreover, a systematic review study illustrated that the mean age of the complicated group is 44 years old (from 3 to 81 years old), and the total amount of white blood cell, especially that of lymphocyte is one of the most important factors affecting the detection of complicated appendicitis [16]. In a broader perspective, almost all our results are similar as the recently medical literature published worldwide. The consistency of our study findings indicated the clinical relevance of our models.

With regards to models trained and validated, there were no significant differences in the accuracy levels and AUCs between unadjusted data and adjusted data. Good to excellent performance of these models indicated their potential in identifying complicated appendicitis. In our study, there was an imbalance in data where the ratio of complicated and uncomplicated appendicitis groups was relatively low. This imbalance could cause false evaluation of the models [20]. The synthetic minority over-resampling (SMOTE) used in our study has been proven to have advantages over other techniques such as the over-resampling methods in fitting the imbalanced data. In this study, accuracy and AUC were used to evaluate the models fitted. While accuracy is a commonly use metric and is more understandable, AUC is preferred to accuracy for imbalanced data. However, after adjusting for imbalanced data using the SMOTE, there is almost no difference between these two metrics. It appeared in our study that GB had the best properties with high values of accuracy and AUC regardless of imbalance adjustment. The accuracy is one of the most common metrics for classification which estimates the probability of the true value label class thanks to the overall effectiveness, while the AUC includes the value of the function of sensitivity and specificity [21].

Several implications can be learned from our study. First, the relatively low prevalence of complicated appendicitis found in this study among patients who underwent a laparoscopic appendectomy at a city hospital highlights the high probability of misclassification of complicated appendicitis. As suggested by Bhangu et al. [4] and Khorram-Manesh et al. [12] patients with noncomplicated appendicitis can be treated with internal medicine, and appendectomy should only be indicated for patients with complicated appendicitis. This hypothesis is underpinned by the absence of a routine CT scan. Second, despite the absence of a CT scan, the inclusion of features in our ML models is to ensure the feasibility of this approach, especially for primary care settings. Coupled with the advantages of ML and models built, our study can be easily deployed and used in similar resource-limited settings. Third, although the study was conducted in a city hospital, the proportion of data used in the analysis was low compared to the total number of patients eligible for this study due to missing data. High data quality remains a big challenge in resource-limited settings. For example, in Vietnam, many hospitals do not have an electronic medical record system. Some hospitals have an electronic medical record system but such system is not optimal. Moreover, data entry is not standardized and most data are stored in the form of text. To take full advantage of artificial intelligence and ML in healthcare, these issues should be addressed in all hospitals.

Our study has several limitations. First, the sample size is relatively small for this type of study and data were collected from a single hospital. This limits our study's generalizability. Further studies are needed to confirm our study findings. Moreover, the rate of complicated appendicitis cases is relatively low, while the qualitative of data is not enough as our expectation. Although our models showed the good to great results, they should be used as a screening tool, not a diagnosis. The actual diagnosis still needs to be decided by specialists and other associated clinical evidence.

## 5. Conclusions

Machine learning approaches including SVM, DT, logistic, KNN, ANN, and GB have a high level of validity in classifying patients with complicated appendicitis and patients without complicated appendicitis. Among these, GB had the highest level of validity and should be used or further validated. Although further studies are needed to confirm our findings in different settings and populations, the accuracy found in our study indicates the beneficial potentials of machine learning techniques in a clinical setting in general and in the diagnosis of complicated appendicitis in particular.

## Figures and Tables

**Table 1:** Data structure used in data analysis.

Variable	Type	Unit	Description
Age	Numeric	Year	Range: 11 - 91
Gender	Binary		1=male; 0=female
White blood cell count (WBC)	Numeric	10^3^ cell/mm^3^	Range: 1.80 - 29.21
Neutrophil	Numeric	10^3^ cell/mm^3^	Range: 1.58 - 27.47
Lymphocyte	Numeric	10^3^ cell/mm^3^	Range: 0.18 - 8.10
Neutrophil lymphocyte ratio (NLR)	Numeric		Range: 0.40 - 65.04
C-reactive protein (CRP)	Numeric	mg/L	Range: 0.2 - 420.3
Diameter of appendix on ultrasound	Numeric	mm	Range: 1 - 80
Appendix position on ultrasound	Binary		1=right lower abdominal quadrant; 0=other positions
Infiltration on ultrasound	Binary		1=yes; 0=no
Abdominal fluid on ultrasound	Binary		1=yes; 0=no
Complicated appendicitis	Binary		1=yes; 0=no

**Table 2:** Characteristics of patients with complicated appendicitis and without complicated appendicitis.

Criteria	Total (*N* = 1950)	Complicated appendicitis		
		Yes *N* = 483 (24.8%)	No *N* = 1467 (75.2%)	Odds ratio (95% confidence interval) ^∗^
Age (year) (mean, standard deviation)	37.3 ± 15.9	40.6 ± 17.3	36.2 ± 15.2	1.02 (1.01 - 1.02)
Gender (*n*, %)				
Male	678 (45.0)	233 (48.2)	652 (44.4)	1.17 (0.95 – 1.43)
Female	826 (55.0)	250 (51.8)	815 (55.6)	Ref
White blood cell count (WBC) (10^3^ cell/mm^3^) (mean, standard deviation)	14.0 ± 3.9	14.4 ± 4.3	13.8 ± 3.8	1.04 (1.01 - 1.06)
Neutrophil (10^3^ cell/mm^3^) (mean, standard deviation)	11.4 ± 3.9	11.9 ± 4.0	11.2 ± 3.8	1.05 (1.02 - 1.08)
Lymphocyte (10^3^ cell/mm^3^) (mean, standard deviation)	1.6 ± 0.7	1.5 ± 0.7	1.7 ± 0.7	0.61 (0.52 - 0.72)
Neutrophil lymphocyte ratio (NLR) (median, interquartile range)	7.0 (4.5 - 11.0)	8.2 (5.3 - 12.5)	6.8 (4.4 - 10.8)	1.03 (1.01 - 1.05)
C-reactive protein (CRP) (mg/L) (median, interquartile range)	14.2 (4.6 – 44.6)	39.8 (10.9 - 105.0)	11.0 (3.8 - 28.8)	1.02 (1.01 - 1.02)
Diameter of appendix on ultrasound (mm) (mean, standard deviation)	9.3 ± 3.0	10.0 ± 2.9	9.1 ± 3.0	1.13 (1.09 - 1.18)
Appendix position on ultrasound (*n*, %)				
Right lower abdominal quadrant	1498 (76.8)	322 (66.7)	1176 (80.2)	0.49 (0.39 - 0.63)
Other positions	452 (23.2)	161 (33.3)	291 (19.8)	Ref
Infiltration on ultrasound (*n*, %)				
Yes	1513 (77.6)	368 (76.2)	1145 (78.0)	0.90 (0.70 - 1.16)
No	437 (22.4)	115 (23.8)	322 (22.0)	Ref
Abdominal fluid on ultrasound (*n*, %)				
Yes	511 (26.2)	172 (35.6)	339 (23.1)	1.84 (1.46 - 2.31)
No	1439 (73.8)	311 (64.4)	1128 (76.9)	Ref

^∗^Odds ratio and 95% confidence interval were from traditional logistic regression.

**Table 3:** Characteristics of machine learning models to classify complicated appendicitis.

Model and parameters used	Description	Imbalance unadjusted data		Imbalance adjusted data	
		*N* = 1950		*N* = 2934	
		AUC	Accuracy	AUC	Accuracy
SVM					
Linear	Liner function	0.711	0.752	0.730	0.655
Gaussian	Radial basis function	0.699	0.798	0.754	0.666
Sigmoid	Sigmoid function	0.627	0.726	0.637	0.596
Polynomial	Polynomial function	0.728	0.805	0.791	0.726
DT					
Gini	Gini impurity	0.574	0.689	0.719	0.719
Entropy	Information gain - entropy	0.609	0.697	0.758	0.758
Logistic					
NCG	Newton -CG	0.711	0.805	0.737	0.675
LBFGS	Limited-memory Broyden-Fletcher-Goldfarb-Shanno	0.711	0.805	0.737	0.675
Lib	Liblinear	0.714	0.803	0.789	0.729
SAG	Stochastic average gradient descent	0.711	0.805	0.737	0.675
SAGA	Stochastic average gradient accelerated	0.714	0.802	0.789	0.726
KNN					
Uniform	Uniform weights	0.680	0.779	0.815	0.734
Distance	Distance weights	0.672	0.776	0.831	0.741
ANN					
GD_a	Gradient descent, identity	0.732	0.807	0.809	0.743
GD_b	Gradient descent, logistic	0.713	0.802	0.803	0.737
GD_c	Gradient descent, tanh	0.734	0.805	0.81	0.742
GD_d	Gradient descent, ReLU	0.748	0.807	0.826	0.766
SGD_a	Stochastic gradient descent, identity	0.692	0.776	0.690	0.632
SGD_b	Stochastic gradient descent, logistic	0.681	0.752	0.614	0.535
SGD_c	Stochastic gradient descent, tanh	0.695	0.774	0.691	0.633
SGD_d	Stochastic gradient descent, ReLU	0.688	0.752	0.689	0.631
LBFGS_a	Limited-memory BFGS, identity	0.744	0.802	0.811	0.750
LBFGS_b	Limited-memory BFGS, logistic	0.641	0.687	0.820	0.755
LBFGS_c	Limited-memory BFGS, tanh	0.644	0.704	0.853	0.776
LBFGS_d	Limited-memory BFGS, ReLU	0.707	0.776	0.838	0.754
GB					
GB1	Friedman MSE, sqrt, deviance	0.741	0.812	0.887	0.823
GB2	Friedman MSE, log, deviance	0.753	0.810	0.890	0.820
GB3	Friedman MSE, deviance	0.746	0.802	0.887	0.825
GB4	Friedman MSE, sqrt, AdaBoost	0.771	0.815	0.888	0.818
GB5	Friedman MSE, log, AdaBoost	0.750	0.810	0.894	0.821
GB6	Friedman MSE, AdaBoost	0.756	0.810	0.887	0.820
GB7	MSE, sqrt, deviance	0.745	0.807	0.89	0.821
GB8	MSE, log, deviance	0.743	0.810	0.886	0.814
GB9	MSE, deviance	0.740	0.803	0.887	0.824
GB10	MSE, sqrt, AdaBoost	0.752	0.809	0.889	0.808
GB11	MSE, log, AdaBoost	0.745	0.812	0.89	0.818
GB12	MSE, AdaBoost	0.755	0.812	0.887	0.820

## Data Availability

The data used to support the findings of this study are available from the corresponding author upon request.
